# Structure and function of Parkin E3 ubiquitin ligase reveals aspects of RING and HECT ligases

**DOI:** 10.1038/ncomms2982

**Published:** 2013-06-17

**Authors:** B.E. Riley, J.C. Lougheed, K. Callaway, M. Velasquez, E. Brecht, L. Nguyen, T. Shaler, D. Walker, Y. Yang, K. Regnstrom, L. Diep, Z. Zhang, S. Chiou, M. Bova, D.R. Artis, N. Yao, J. Baker, T. Yednock, J.A. Johnston

**Affiliations:** 1 Elan Pharmaceuticals, 180 Oyster Point Boulevard, South San Francisco, California 94080, USA; 2 SRI International, 333 Ravenswood Avenue, Menlo Park, California 94025, USA; 3 These authors contributed equally to this work

## Abstract

Parkin is a RING-between-RING E3 ligase that functions in the covalent attachment of ubiquitin to specific substrates, and mutations in Parkin are linked to Parkinson’s disease, cancer and mycobacterial infection. The RING-between-RING family of E3 ligases are suggested to function with a canonical RING domain and a catalytic cysteine residue usually restricted to HECT E3 ligases, thus termed ‘RING/HECT hybrid’ enzymes. Here we present the 1.58 Å structure of Parkin-R0RBR, revealing the fold architecture for the four RING domains, and several unpredicted interfaces. Examination of the Parkin active site suggests a catalytic network consisting of C431 and H433. In cells, mutation of C431 eliminates Parkin-catalysed degradation of mitochondria, and capture of an ubiquitin oxyester confirms C431 as Parkin’s cellular active site. Our data confirm that Parkin is a RING/HECT hybrid, and provide the first crystal structure of an RING-between-RING E3 ligase at atomic resolution, providing insight into this disease-related protein.

## Abstract (web summary)

The Parkinson’s disease-associated protein Parkin regulates the fate of damaged mitochondria by ubiquitinating mitochondrial substrates. Riley *et al*. present the crystal structure of the Parkin-R0RBR domain, providing new insight into the catalytic mechanism of the enzyme.

## Graphical abstract

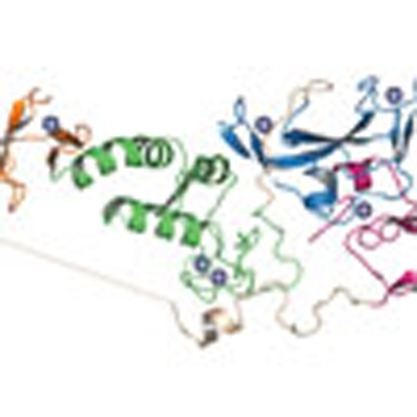

Posttranslational modification of proteins by ubiquitin is a fundamental cellular mechanism that regulates protein stability and activity and underlies a multitude of functions, from almost every aspect of biology. The covalent attachment of ubiquitin to specific protein substrates is achieved through the action of E3 ubiquitin ligases. These ligases comprise over 500 different proteins and are categorized into multiple classes defined by the structural element of their E3 functional activity. Specifically, both HECT and RING ligases transfer an activated ubiquitin from a thioester to the ε-amino acid group of a lysine residue on a substrate; however, HECT ligases have an active site cysteine that forms an intermediate thioester bond with ubiquitin, while RING ligases function as a scaffold to allow direct ubiquitin transfer from the E2 to substrate. Recent evidence suggests that a subfamily of RING ligases, the RING-between-RING (RBR) family, may contain a catalytic cysteine residue^1^,^2^ in addition to a canonical RING domain.

Parkin has been proposed to function as a RBR ligase such that it encompasses both of the major classes of E3 ligase in one protein. Specifically, it may function with both a catalytic cysteine and a classical RING motif for binding E2. Although recent work has established that Parkin has four RING domains, coordinating eight zinc (Zn) molecules^3^, the exact residues coordinating these Zn atoms, and the organization of each of the RING domains with respect to each other are not known. Parkin has been described to have latent activity that can be activated with carbonyl cyanide 3-chlorophenylhydrazone (CCCP) in cells, although it is not completely known how the latent state becomes activated at the molecular level^4^, and whether or not purified Parkin protein contains a similar latent state. Regulation of Parkin activity by phosphorylation has been described^5^, but the subsequent molecular events post-phosphorylation are not understood. Finally, although catalytic networks have been investigated for E3 ligases^6^, it is not yet clear whether they function with a classic triad/dyad-based mechanism, or whether catalysis occurs through a hydrogen-bonding network. For deubiquitinating enzymes (DUBs), it has been demonstrated that the cleavage of ubiquitin from a substrate occurs through a classic triad/dyad mechanism, utilizing a critical catalytic cysteine residue, and a histidine residue in close proximity^7^,^8^,^9^,^10^,^11^.

To gain insight into the domain organization of Parkin, and regulation of Parkin ligase activity, we sought to obtain the crystal structure of Parkin at high resolution. Our 1.58 Å structure reveals that Parkin forms a relatively compact overall structure with multiple unpredicted domain interfaces. These interfaces form the basis for understanding a latent and activated state for Parkin, as well as provide insight into the role of the active site cysteine, C431, and the network of residues in proximity to C431 that facilitate catalysis. Our data support the suggestion that RBR ligases function with both RING and HECT-like mechanisms, and provides a blueprint for future mechanistic and functional studies.

## Results

### Overall structure and individual domains

**Figure 1:**
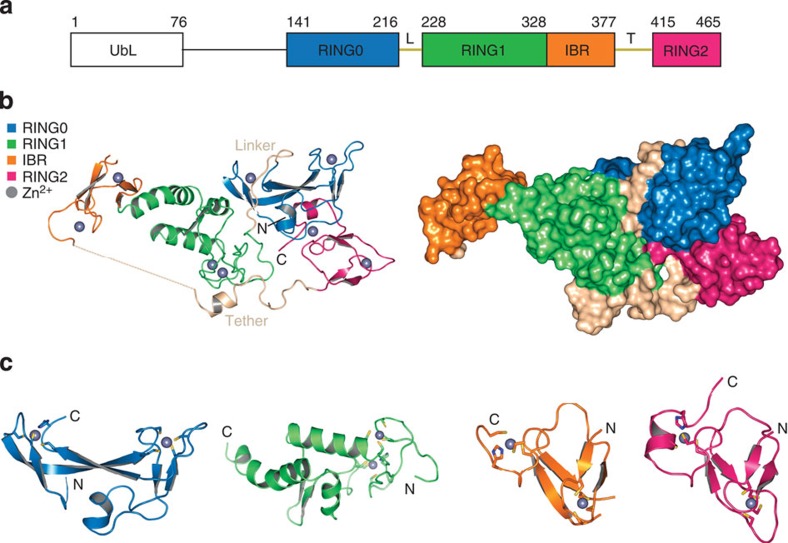
Overall Parkin domain organization and RING structures. (**a**) Schematic diagram of Parkin indicating linear domain organization and structural domain boundaries. L denotes linker and T, the tether. (**b**) Overall ribbon diagram of R0RBR (left) and overall surface structure (right). (**c**) View of individual RING domains. See Supplementary Fig. S2 for further details.

To gain insight into the RBR class of E3 ligase, we grew crystals of Parkin-R0RBR (residues 141–465) that includes RING 0 (R0) and the RBR domains (Fig. 1a). The structure was determined by analysis of multiwavelength anomalous diffraction data using the signal from Zn ions bound by the individual RING domains and was refined against high resolution data to 1.58 Å (Supplementary Fig. S1, Supplementary Table S1–S2). Coordinates and structure factors have been deposited in the RCSB protein data bank under accession codes 4I1F (Parkin-R0RBR-P223) and 4I1H (Parkin-R0RBR-S223). We solved two structures of Parkin to determine if there were any notable structural differences between the sequence of Parkin originally reported (containing P223) and the updated sequence (S223). Overall the two structures were extremely similar (Supplementary Fig. S2); however, the loop containing S223 was visible in Parkin-R0RBR but not in the P223 structure. This residue has had considerable discussion (http://mutview.dmb.med.keio.ac.jp/Parkin-seq/message.html) although there has been no mitochondria targeting sequence identified in that region (Narenda and Youle, personal communication). Aspects discussed in this paper were indistinguishable for Parkin with S or P at position 223; The structure discussion focuses on the S223 protein. Each RING domain binds two Zn ions and resolves discrepancies in the literature regarding Zn coordination^3^,^12^,^13^ (Fig. 1c, Supplementary Fig. S3). The R0 domain (residues 141–216) is a previously unobserved domain fold (based on Dali)^14^, while R1 (residues 228–328) shows the classical cross-brace arrangement and Zn coordination of canonical RING domains^15^,^16^ (Supplementary Fig. S3b). The IBR domain is similar to the published NMR structure^17^ (Supplementary Fig. S3c). The R2 domain of Parkin most closely resembles IBR domains, and is very similar to HOIP IBR domain (Supplementary Fig. S3c). Conversely, R2 differs significantly from the closely related HHARI R2 NMR structure^18^, and it will be interesting to compare additional RBR protein structures as they become available^19^.

**Figure 2:**
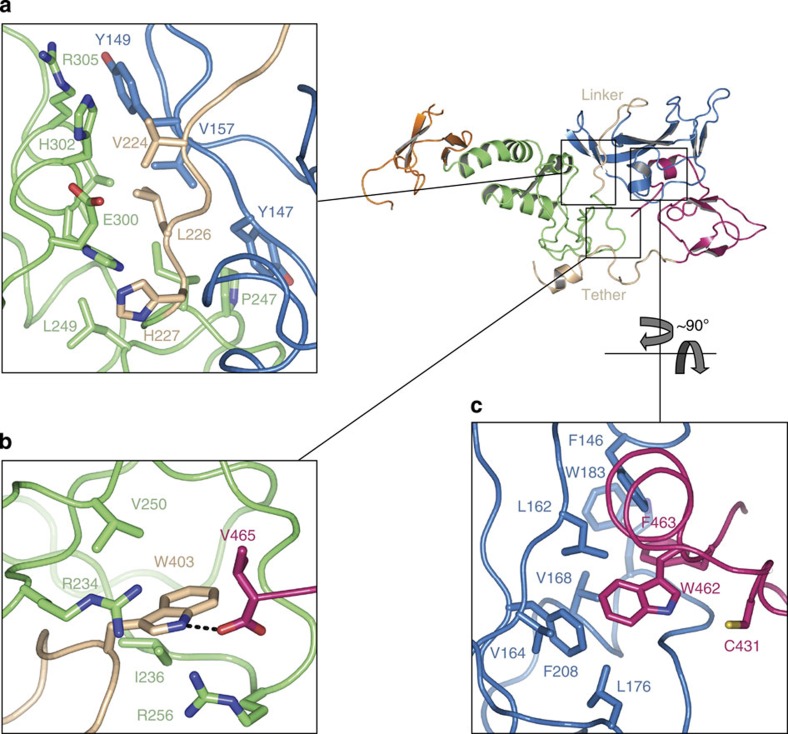
R0RBR is assembled into two compact domain groups separated by linkers. (**a**) The R0 (blue) and R1 (green) interface is relatively hydrophilic and separated by the R0–R1 linker (beige), suggesting this area may have some structural flexibility. (**b**) The tether (beige) residue W403 sits in a hydrophobic pocket on R1 and may serve as a ‘pin’ to anchor the two turn helix of the tether to R1. W403 also forms a hydrogen bond with the terminal carboxylate of V465 (pink). R256 is the site of a human PD mutation. (**c**) The R0 (blue) domain forms a hydrophobic interface with the catalytic domain R2 (pink), inserting residues W462 and F463 into the hydrophobic core of R0. The catalytic cysteine, C431, is adjacent to this interface.

The overall R0RBR structure reveals two compact domain groups separated by two linkers (Fig. 1b). One domain is comprised of an association between RING1 (R1) and IBR formed by a small hydrophobic patch at their interface boundary. As R1 has a canonical RING structure, it can be visualized as the binding site for E2 based on the structure of other RING ligases (Supplementary Fig. S4). The other domain is formed by close association of R0 and R2, involving a total of ~1,330 Å (ref. 2) surface area between the C-terminal region of R2 and the hydrophobic core of R0 (Figs 1b, 2c). R2 contains the proposed catalytic cysteine (C431) that is near the hydrophobic interface with R0. The R0:R2 interface is unique to Parkin as it involves sequences within R0 as well as the C-terminus of R2 that are distinct from other RBR ligases. The R0–R1 linker region between the two major domains has a coil conformation and resides in a relatively hydrophilic interface (Fig. 2a), suggesting that this may be an area of potential structural flexibility. The IBR-R2 linker region (which we refer to as the tether: residues 378–414) is 37 residues long. Most of the beginning of the tether (14 residues) is disordered, while the latter part runs across the surface of R1 (Fig. 2b, Supplementary Fig. S4c). The tether forms significant interactions with R1, but is also an area of potential flexibility. A two turn helix (residues 394–401) packs against R1 and the nearby residue W403 may serve as a ‘pin’ to anchor the tether to R1 and associate it with R2 (Fig. 2b). The side chain of W403 sits in a hydrophobic pocket formed by several R1 residues and forms a hydrogen bond with the terminal carboxylate of V465 (Supplementary Fig. S4c) a residue only found in mammals (Supplementary Fig. S7b). As described above, R1 is a likely E2-binding site on Parkin and the position of the tether has the potential to regulate this interaction (Fig. 2b, Supplementary Fig. S4).

### Investigation of a catalytic mechanism for Parkin

**Figure 3:**
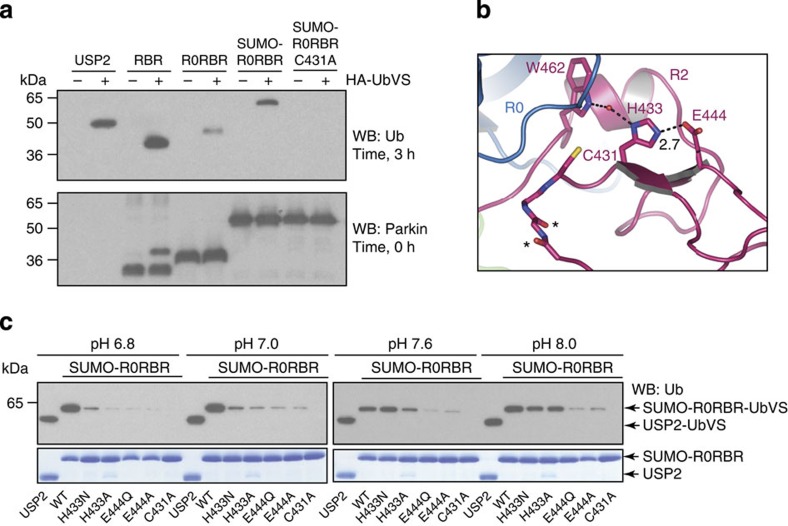
Catalytic machinery of Parkin. (**a**) The activity-probe HA-Ub-VS was incubated with various Parkin constructs (or USP2 control) to determine intrinsic Parkin enzymatic activity. Reactions were allowed to proceed for 3 h, or for the Parkin blot (loading control) samples were removed at time zero (t0) hours and terminated by the addition of SDS-loading dye. For RBR samples, the reaction proceeds so quickly that even at time zero, the reaction is observed. (**b**) The potential catalytic triad residues C431, H433 and E444 are misaligned. H433 is engaged in a water-mediated hydrogen bond with W462 and is ~5.1 Å from C431. A GG-C431 motif is present (asterisks), which could serve as a classical oxyanion hole during catalysis. (**c**) Parkin probe reactivity requires elements of a classical catalytic triad. Ub-VS probe reactivity was assessed in various Parkin mutants over a range of pH.

To interrogate the accessibility of Parkin’s catalytic machinery without the confounding factors that result from the interactions of E2 in a transthiolation reaction, we used the activity-probe ubiquitin-vinyl sulfone (Ub-VS). Ub-VS is a specific probe that will covalently modify the active site cysteine of DUBs and HECT ligases through specific recognition of ubiquitin and the oriented positioning of the VS moiety^20^,^21^. The R0RBR domain of Parkin weakly reacted with Ub-VS, while the RBR domain of Parkin produced a ~8-kDa shift in molecular weight indicating that the RBR domain of Parkin is more reactive to probe than the R0RBR. N-terminally SUMO-tagged R0RBR was also active in this assay (Fig. 3a). These results suggest that removal or modification of R0, which is closely aligned with R2, may allow conformational changes near the active site to facilitate probe reactivity. The intensity of Ub-VS probe labelling has been suggested to correlate with the functional state of the active site of the enzyme^21^ and in support of this idea we found that autoubiquitination activity was consistent with probe binding (Fig. 3a, Supplementary Fig. S5b). Furthermore, these results are consistent with recent published work demonstrating greater levels of activity for RBR compared to R0RBR Parkin constructs^22^. Using mass spectrometry we confirmed the Ub-VS was attached only to Parkin’s C431 (Supplementary Fig. S5a), and confirmed labelling of Parkin was specific for Ub-VS as other ubiquitin-like VS moieties did not robustly label Parkin (Supplementary Fig. S6a). Although there is an example of a ligase (A20) that also has DUB activity^23^, this is not the case for Parkin (Supplementary Fig. S6b) and is not likely to be the reason that Parkin can be labelled with Ub-VS probe.

In transthiolation reactions, the active site cysteine needs to be activated for nucleophilic attack of the E2~ubiquitin thioester carbonyl bond^24^. Activation of the cysteine and stabilization of the resulting tetrahedral intermediate require the presence of characteristic elements found in cysteine proteases or DUBs: an activating catalytic dyad^8^ or triad^7^,^9^,^10^,^11^, and an oxyanion hole framed by backbone or side chain hydrogen bond donors^25^. Examination of the residues surrounding Parkin’s active site C431 revealed putative active site triad residues consisting of C431, H433 and E444 (Fig. 3b). These residues are conserved across all species of Parkin examined (Supplementary Fig. S7b), and mutation of H433 and E444 significantly disrupted Ub-VS probe reactivity at neutral pH (Fig. 3c), and reactivity was completely eliminated by mutation of C431 (Fig. 3a). The role of histidine within a catalytic dyad or triad is to function as a base that deprotonates the cysteine for activation—a role that can potentially be obviated by elevated pH. Consistent with this mechanism, we found that although H433A or H433N demonstrated little probe reactivity at neutral pH, probe labelling was restored with pH titration (Fig. 3c). As is common for DUBs^7^,^11^ in our structure C431 and H433 are not well aligned for catalysis and implies that a conformational rearrangement must occur for catalysis to take place (Fig. 3b).

### Latent activity and catalytic residues in cells

**Figure 4:**
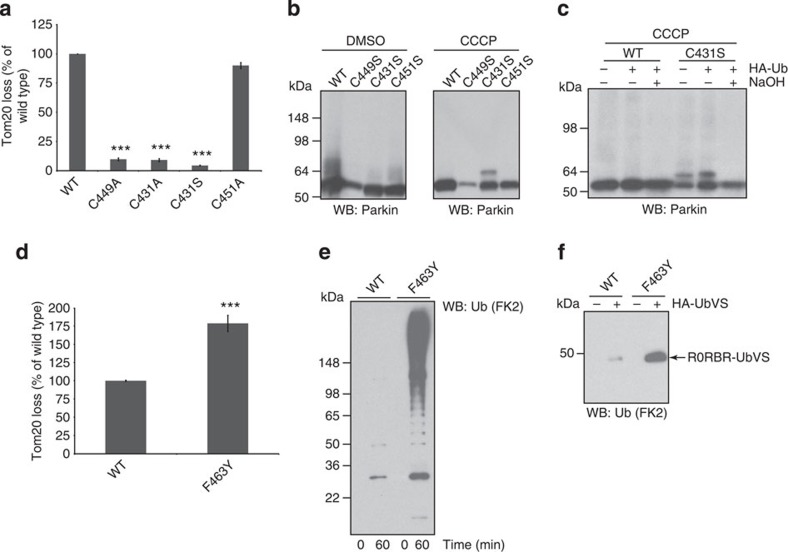
Mitochondrial stress activates Parkin and drives exposure of the active site C431. (**a**) Parkin active site mutants C431S/C431A compromise Parkin’s ability to decrease cellular Tom20 levels. Mutations in Parkin’s Zn binding residue C449 also inhibited Parkin’s ability to function in this assay. Full-length wild-type Parkin-induced Tom20 loss after CCCP treatment. DMSO treated was set to 100%. Data shown are representative of three-independent experiments (error bars represents s.e.m.). The significance levels were determined using the heteroscedastic Student’s *t*-test with two-tailed distribution. Triple asterisk denotes *P*≤0.005 (C449A, *P*=0.00006; C431A, *P*=0.00004; C431S *P*=0.001). (**b**) Western blot showing formation of ~8 kDa Parkin immunoreactive species during mitochondrial stress (CCCP) only in cells expressing full-length Parkin C431S. (**c**) Western blot showing the ~8 kDa Parkin immunoreactive species is sensitive to sodium hydroxide treatment indicative of ubiquitin oxyester formation on full-length Parkin C431S. (**d**) Enhanced cellular activity of full-length Parkin F463Y compared to full-length wild-type Parkin. Data shown are representative of three-independent experiments (error bars represent s.e.m.). The significance levels were determined using the heteroscedastic Student’s *t*-test with two-tailed distribution. Triple asterisk denotes *P*≤0.005 (F463Y, *P*=0.002). (**e**) Autoubiquitination of R0RBR F463Y is increased compared with wild-type R0RBR. (**f**) Increased HA-Ub-VS probe labelling of R0RBR F463Y compared with wild-type R0RBR.

In cells, Parkin has been shown to have an important role in Parkin-catalysed degradation of mitochondrial protein, following treatment with mitochondrial toxins, such as CCCP^26^. To assay functional activity of C431 in cells, we examined Tom20 loss as a measure of Parkin-catalysed degradation of mitochondrial protein, following CCCP treatment. Although Mitofusions and other mitochondrial proteins have been reported to be Parkin substrates^27^,^28^,^29^,^30^, in our hands Tom20 loss was a highly reproducible Parkin-dependent event after CCCP treatment. The active site cysteine mutants, C431S and C431A, while soluble and well-behaved (Supplementary Fig. S8a), were unable to function in this cellular assay (Fig. 4a), consistent with the utilization of Parkin’s active site C431 during mitochondrial stress. Moreover, we were able to demonstrate that the C431S mutant formed an ubiquitin oxyester only in the presence of CCCP, directly supporting C431 as an active site residue in cells (Fig. 4b and Supplementary Fig. S8b) and that Parkin has latent activity that can be activated by CCCP^4^. Although, there is the formal possibility that C431 is also involved in translocation of Parkin to mitochondria^31^. Mutation of H433 and E444 in our cellular assay revealed a requirement for H433, but no requirement for E444 (Supplementary Fig. S8c) even though neither mutation affected protein levels. Why E444 is dispensable in cells is not clear, although Parkin may have a binding partner in cells that provides the role of the Glu in positioning the His, or the pH at the mitochondria membrane may be such that the E444 is dispensable for deprotonation of C431. Although the Cys, His and Glu residues are completely conserved across all species of Parkin (Supplementary Fig. S7b), the motif is not conserved across all RBR ligases, (Supplementary Fig. S7a) and other RBR ligases may use a different catalytic mechanism.

### Critical phenylalanine differs from HECT ligases

Inspection of the extreme C-terminus of Parkin revealed a conserved phenylalanine (F) at the −3 position (Supplementary Fig. S7b), F463, and Phe at the −5 to −3 position has been described as a critical determinant for positioning of the incoming substrate lysine in HECT ligases^6^,^32^. Mutation of F463 to tyrosine (Fig. 4d) enhanced the activity of Parkin in cells for Tom20 loss as well as dramatic increases in autoubiquitination activity and Ub-VS probe binding of R0RBR *in vitro* (Fig. 4e). This suggests that the conserved F463 at the extreme C-terminus of Parkin is likely to serve in a distinct capacity from the extreme C-termini of HECT ligases structurally described thus far. In our structure, F463 is involved in critical hydrophobic interactions predicted to contribute to integrity of R0:R2 interface (Fig. 2c). Coupled with the data above, this result suggests that the R0:R2 interface defined in the Parkin structure is important in regulation of Parkin’s active site. In fact, mutation of any of the hydrophobic residues that comprise the R0:R2 interface results in increased autoubiquitination activity (Supplementary Fig. S8c). Thus, the −3 Phe in Parkin functions distinctly from similarly spaced residues in other HECT ligases, and suggests that it is possible to increase Parkin activity through changes in the integrity of the R0:R2 interface.

### PD-associated mutations map to Parkin functional areas

**Figure 5:**
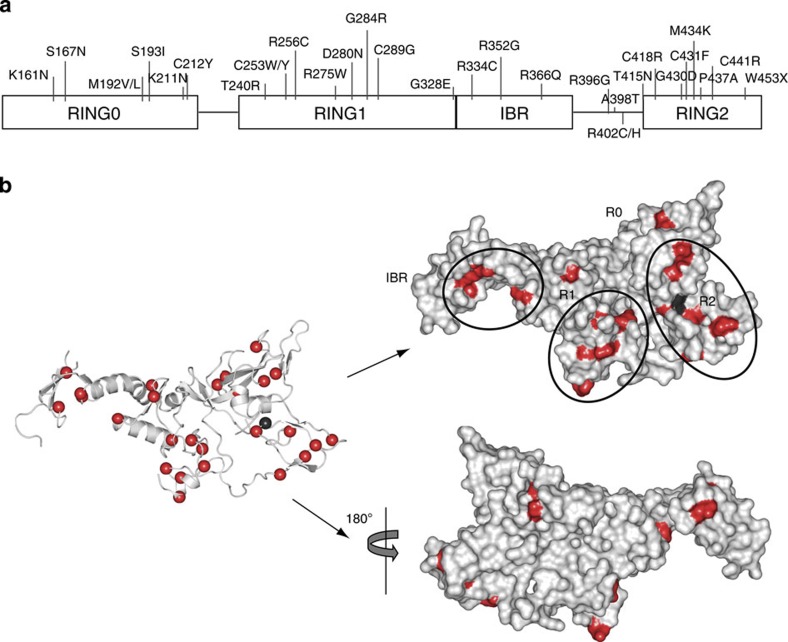
Human genetic PD mutations mapped on Parkin-R0RBR. (**a**) Schematic diagram of Parkin-R0RBR indicating residues that can be mutated in PD. (**b**) R0RBR ribbon representation (left) and space filling model (right) with two 180° views. PD mutations are shown in red and the catalytic cysteine C431 is shown in black. One face of Parkin has a higher number of mutations than the other face. Several areas contain higher densities of mutations, and these regions are circled. These functional regions include the area near the R1:IBR interface, the putative E2-binding site, and the area around the catalytic C431.

We selected twenty-eight (of the approximately seventy) human genetic mutations in Parkin^15^,^28^,^33^,^34^,^35^,^36^,^37^ that represented an unbiased distribution throughout the various Parkin domains (in R0RBR) for mapping analysis in our structure. Examination of these mutations in Parkin revealed that ten mapped to residues directly involved or closely aligned with Zn coordination. The other mutations are present in each of the four domains and the tether (Fig. 5, Supplementary Table S3), and can be roughly grouped into two functional regions; E2 binding and the area surrounding the catalytic cysteine. There are three PD mutations (R396G, A398T, R402C/H) that occur in the linker closely associated with R1 in the region of the short α-helix of the tether, and A398T would be predicted to directly perturb the interaction with R1 and have implications for E2 binding. Furthermore, the R1 mutation T240R may interrupt E2 binding as was reported experimentally^38^. Mutations in the IBR domain and at the R1:IBR interface could disrupt structural integrity and E2 binding. Finally, numerous mutations occur around the catalytic C431, these mutations might disrupt substrate/cofactor binding or catalytic efficiency.

## Discussion

We have solved the structure of Parkin-R0RBR at high resolution. Overall, our structure demonstrates characteristics of RING and HECT ligases, as has been suggested by previous biochemical analysis of HHARI^2^. Analysis of this structure reveals several new aspects of the Parkin protein including: novel RING structures for R0 and R2, insight into the catalytic activity at the molecular level, unpredicted interfaces between domains and a clustering of human PD mutations that were not indicated from linear mapping studies.

The individual RING domains for Parkin have been the subject of much debate, in regards to the specific residues that coordinate Zn ions, as well as their relationship to canonical RING cross-brace structures defining classical E2-binding domains. R1 is the only RING domain of Parkin that demonstrates a typical E2 binding motif, with a cross-brace structure that defines this domain^15^,^16^. R0 is a novel domain structure, but is more similar to Zn-finger domains than to E3 RING domains (Fig. 1d). The IBR domain is largely as predicted from high-quality NMR studies of the isolated IBR domain from Parkin^17^. RING2 is similar to the IBR domain of Parkin and HOIP (Supplementary Fig. S3c). However, neither resembles canonical RING domain motifs, as they do not have a cross-brace structure. These findings call into question whether the RING nomenclature is actually appropriate for the R0, IBR and R2 domains.

Previous work defining RBR ligases as RING/HECT hybrids predicted a HECT-like catalytic cysteine residue in the R2 domain^1^,^2^,^39^. Although the R2 domain does not resemble a typical HECT structure, our analysis confirmed that there is a catalytic cysteine at C431. As discussed above for RING nomenclature, it may be useful to reassess the use of the ‘HECT’ term for ligases with catalytic cysteines. Similar to what we have found with Parkin, a series of bacterial ligases have recently been described that function through a catalytic cysteine residue, but bear no sequence or structural similarity to HECT domains^40^,^41^,^42^. Thus, bacterial ligases, RBR ligases and HECT ligases function through a catalytic cysteine, but are structurally and sequence-wise distinct.

Mechanistically, we identified residues in the catalytic core that may function with C431 to promote catalysis. Mutational studies of H433 and E444 demonstrated that H433 is required to promote catalytic activity through C431 *in vitro* and in cells. Whether this finding is representative of a catalytic triad/dyad mechanism as has been demonstrated for DUBs^7^,^8^,^9^,^10^,^11^ or whether it represents a mechanism that functions through an hydrogen-bonding network with other residues remains to be determined through more extensive mutational analysis. However, our use of the Ub-VS probe clearly demonstrates that H433 is involved in the transthiolation step from E2 to E3 at C431. Importantly, the access to C431 may be restricted, and serve as a means to regulate Parkin activity (see below).

The R0RBR structure reveals that the molecule is folded in half with the N- and C-termini forming an extensive interface that comprise two compact domain groups separated by two linkers (Fig. 1b). One domain group is comprised of an association between R1 and IBR, the other domain group is formed by a close association of R0 and R2. The R1:IBR domain group contains the putative E2-binding site, which appears to be obstructed by a portion of the IBR, that we have designated the ‘tether’. Mutational analysis of this tether region, including W403, is likely to yield important insight into activation of Parkin in regards to E2 access. A key feature of the R0:R2 domain group is the positioning of the C-terminus of Parkin R2 into a hydrophobic region on R0. The R0:R2 interface may contribute to regulation of Parkin activation in two ways: By restricting access of the incoming ubiquitin C-terminal Gly-Gly to Parkin C431, and by misalignment of Parkin’s catalytic machinery, namely H433 and C431. Our functional analysis of the R0:R2 interface demonstrated that mutation of individual hydrophobic residues resulted in activation of Parkin autoubiquitination and probe label (Fig. 4 and Supplementary Fig. S8d). As probe label depends upon access to the active site, and appropriate alignment of catalytic residues, this result suggests that R0:R2 interface mutations likely function in one or both of the two ways suggested above. We did not observe increased ubiquitin binding in these mutants by Biacore, suggesting they do not function to increase affinity for Ub. In our structure, H433 is involved in water-mediated hydrogen bonding with W462 and is not available to deprotonate C431 (Fig. 3c), representing an inactive state of the enz**y**me through misalignment of the catalytic residues.

Notably, mutation of F463 does not result in loss of Parkin activity. In other HECT ligases, a similarly spaced phenylalanine is thought to position the E3 ligase thioester-bound ubiquitin for transfer to substrate, and is critical for activity^6^,^32^. Thus, although Parkin exhibits catalytic cysteine activity similar to HECT ligases, it is also functionally distinct, as the −3 Phe appears to function in a different capacity. Finally, the movement within the interface between the two large domains formed by R1:IBR and R0:R2 may provide a way in which to juxtapose the catalytic cysteine of UbcH7 (bound to R1) with the active site C431 in the R2 of Parkin. In our current model, E2 (UbcH7) bound to Parkin is too distant for transthiolation of C431 to occur (Supplementary Fig. S4). The structure suggests the potential for a conformational change to facilitate transthiolation from UbcH7 to Parkin. We envision this movement to be that of a butterfly motion whereby R1:IBR and R0:R2 move with respect to each other via the flexible linker and tether regions shown in beige (Figs 1b,c,2a, and Supplementary Fig. S4a). However, a full mutational analysis of these interfaces, and a structural determination of Parkin bound to UbcH7 during transthiolation, will be necessary to address this issue. Such studies have been done for other ligases, and have demonstrated linkers that promote and allow for considerable conformational flexibility^43^.

Our structural analysis and mapping of PD mutations of Parkin demonstrates that there are key functional areas on Parkin that are affected by mutations. Largely, the mutations cluster into three groups: (1) Zn coordination residues, likely to affect overall structural stability; (2) predicted E2 binding region, with mutations in the direct binding site for the E2, as well as in regions proximal to the predicted E2 binding site that may affect movement of the tether residues or aspects that are still not yet understood about E2–E3 binding interactions; and (3) the catalytic region around residue C431. This map will be highly useful in future investigational studies of the functional role for these mutations in catalysis, E2 binding, conformational flexibility, activation of a latent state, as well as potential regions for critical binding partners. Functional studies have also defined key features of isolated Parkin mutations^4^,^28^,^44^, and some of these mutations may help identify regions of Parkin that are important for localization to mitochondria. The fact that the mutations mapped in this study largely occur on one face of the molecule is suggestive of a specific functionality for a directed orientation relative to other molecules. Before this study, it was very difficult to understand Parkin mutations in an overall picture, but this structural snapshot will provide a map for testing new hypotheses, such as those suggested in Supplementary Table S3.

Our structural, biochemical and cellular data indicate that Parkin functions as a RING/HECT hybrid. Parkin likely binds E2 through a conserved structural motif on a canonical RING domain, and also functions through a HECT-like active site cysteine whose activity can be regulated through interaction between the R0 and R2 domains. Our structure of Parkin-R0RBR will be useful for drug discovery efforts aimed to increase ligase activity, as well as to elucidate the molecular mechanisms of ubiquitination in this new class of E3 ligase.

## Methods

### General reagents and DNA constructs

The DNA template for all Parkin constructs was NCBI reference sequence NM_004562 and NM_004562.1. For bacterial expression full-length Parkin (1–465), R0RBR (141–465) and RBR (238–465) were cloned into Champion pET SUMO vector per the manufacturer’s instructions (Invitrogen). For mammalian expression Parkin (untagged full-length) was cloned into pcDNA3.1. All mutations were created using QuikChange Site-Directed Mutagenesis Kit (Agilent Technologies). For western blotting all antibodies were used at 1:1,000 per the manufactures recommendations. Anti-Parkin Ab (Prk8) was from Sigma. Anti-Parkin Ab (HPA1A) was a rabbit polyclonal Ab that was raised against an N-terminal peptide of Parkin (a.a. 85–96). Anti-GAPDH Ab was from Millipore. Anti-ubiquitin Ab (FK2) was from Enzo Life Sciences. Rabbit anti-Tom20 Ab was from Santa Cruz. Alexa 594 and DAPI were from molecular probes (Invitrogen). Mouse anti-HA Ab was from Covance. CCCP was from Sigma. E1, UbcH7, UbcH8, Ub, Mg-ATP solution were all from Boston Biochem. HA-Ub-VS and all other Ub-like-VS were from Boston Biochem. The anti-FLAG M2 agarose resin and the 3X FLAG peptide were obtained from Sigma-Aldrich. Protein A beads were from Repligen.

### Protein expression

Bacterial expression constructs were transformed into BL21 DE3 *E. Coli* (Invitrogen). Overnight cultures inoculated from fresh colonies were grown in Terrific broth media containing 2% glucose and 50 μg ml^−1^ kanamycin at 37 °C. The following morning overnight cultures were diluted to OD_600_ 0.1 and continued shaking at 37 °C until OD_600_ reached 0.4, flasks were then transferred to 16 °C, upon OD_600_ 0.8–0.9, cultures were induced with 0.1 mM IPTG supplemented with 50 μM zinc chloride and expression was allowed to proceed for 18–20 h at 16 °C. Cells were then harvested by centrifugation and frozen at −80 °C.

### Protein purification

High-performance Ni sepharose, the Mono Q HR 10/10 anion exchange column and the HiLoad 26/60 Superdex 200 column were all from GE Life sciences. FPLC was performed on an ÄKTA FPLC system. UV−Vis absorbance readings were taken on a Nanodrop spectrophotometer. Protein was analysed by SDS−PAGE under denaturing conditions on 10% Bis-Tris NuPAGE gels using MES running buffer (Invitrogen). The extinction coefficients (*ε*) for the denatured proteins were determined from the primary sequence, according to *ε*=5,690 cm^−1^ M^−1^ × (number of trp)+1,280 cm^−1^ M^−1^ × (number of tyr)+120 cm^−1^ M^−1^ × (number of cys-where cys=cystine or disulphide bond)^45^.

For purification of SUMO-Parkin constructs from bacteria, cells were resuspended in buffer A (50 mM Tris pH 8.0, 200 mM NaCl, 10 mM imidazole, 250 μM TCEP and EDTA-free Complete protease inhibitor tablets (Roche)) and lysed using a microfluidizer. The lysate was cleared (45,000 *g*, 25 min, 4 °C) and the supernatant agitated gently with high-performance Ni sepharose (0.625 ml resin per l cell culture) for 1 h at 4 °C. The beads were washed with 10 column volumes of buffer A containing 20 mM imidazole and then washed with 10 column volumes of buffer A containing 40 mM imidazole. The protein was eluted with 10 column volumes of buffer A containing 200 mM imidazole. After elution, the protein was dialysed into 50 mM Tris for 2 h at 4 °C to reduce the salt concentration. The protein was then loaded onto a Mono Q HR 10/10 anion exchange column that had been pre-equilibrated in buffer B (50 mM Tris pH 8.0 and 250 μM TCEP). The column was developed with a gradient of 0–500 mM NaCl over 50 column volumes and the protein was eluted at 113–180 mM NaCl. Collected fractions were then concentrated and injected onto a HiLoad 26/60 Superdex 200 column that had been pre-equilibrated in buffer C (25 mM HEPES pH 8.0, 50 mM NaCl and 1 mM TCEP). The column was eluted with 1.5 CV of buffer C.

For removal of the SUMO tag, purification was as described above except after the Mono Q column protein was incubated with SENP1 (10:1 w/w ratio of protein to SENP1) for 2 h at 4 °C. Following the incubation, 10 mM imidazole was added to the cleavage reaction and the reaction was purified over a high-performance Ni sepharose column (0.625 ml resin per l cell culture). The Ni column was washed with 10 CV of buffer A. Both the wash and the flowthru from the Ni column were collected and injected onto a HiLoad 26/60 Superdex 200 column that had been pre-equilibrated in buffer C (25 mM HEPES pH 8.0, 50 mM NaCl and 1 mM TCEP). The column was eluted with 1.5 CV of buffer C.

### Crystallization and structure determination

Parkin-R0RBR-P223 protein crystals were grown in sitting drops containing 0.3 μl each of 12.5 mg ml^−1^ protein in 25 mM HEPES (pH 8.0), 50 mM NaCl and 1 mM TCEP and a reservoir of 0.1 M HEPES (pH 7.5), 20% PEG 4K, 10% isopropanol, 10 mM BaCl_2_ at 10 °C. Seeding was used to obtain higher quality crystals and crystals generally reached full size in 4–7 days. Crystals were transferred to 15% ethylene glycol in reservoir solution before being flash cooled in liquid nitrogen. The crystals belong to the space group C222_1_ (*a*=86.96 Å, *b*=133.16 Å, *c*=65.39 Å) and contain one molecule per asymmetric unit. Synchrotron X-ray data were collected on a single crystal at a peak/inflection compromise and remote wavelengths in order to measure the Zn anomalous and dispersive signals. Diffraction data were integrated with MOSFLM^46^ and scaled with SCALA^47^. Nine anomalous sites were found by SHELXD^48^ and phases were refined with MLPHARE^47^ (Supplementary Table S1). Solvent flattening against high-resolution data (1.58 Å) collected at 1.1159 Å using DM^49^ resulted in a clearly interpretable electron density map (Supplementary Fig. S1b). The mean figure of merit was 0.217 after MLPHARE and 0.715 after solvent flattering. The model was built manually into this map using the program Coot^50^. One of the anomalous sites was modelled as barium based on the characteristics of the coordinating ligands. The structure was refined against the high-resolution data using REFMAC^51^, and contains 306-aa residues and 267 water molecules. Both structures have been deposited in the protein data bank, pdb codes 4I1F (Parkin-R0RBR-P223) and 4I1H (Parkin-R0RBR-S223).

Parkin-R0RBR-S223 crystals were grown in sitting drops using 0.3 μl each of 10 mg ml^−1^ protein in the same buffer as Parkin-R0RBR-P223 and a reservoir of 0.1 M Tris (pH 6.5), 0.2 M NaCl and 25% PEG 3350 at 10 °C. The crystals grew over 3 days and then were transferred to 10% ethylene glycol in reservoir solution before being flash cooled in liquid nitrogen. Crystals belonged to C222_1_ (*a*=87.11 Å, *b*=133.9 Å, *c*=66.21 Å). Data were collected using a home source Saturn 944 detector and Rigaku MicroMax007HF generator, processed with MOSFLM^46^ and scaled with SCALA^47^. The Parkin-R0RBR-P223 structure was used as a starting model and rebuilt as necessary in Coot^50^, alternating with rounds of refinement to 2.0 Å in REFMAC^51^, and the final model contains 306-aa and 263 waters (Supplementary Table S2).

### Biochemical assays

Parkin autoubiquitination reactions were typically carried out in a 25-μl reaction volume in reaction buffer of 50 mM HEPES, 50 mM NaCl, pH 8.0 for 1 h at 37 °C using E1 (250 nM), E2 (5 μM), ubiquitin (23.5 μM), Mg-ATP solution (10 mM) and Parkin species (0.46 μM). Reactions were terminated by the addition of SDS-loading buffer.

Parkin activity-probe labelling with HA-Ub-VS was as previously reported^20^. Briefly Parkin (5 μg) was incubated with HA-Ub-VS (or other Ub-like VS, Boston Biochem) at 3:1 Parkin:Ub-VS molar ratio or at 1:1 Parkin:Ub-VS ratio for 3 h at room temperature in 50 mM HEPES, 50 mM NaCl, over a range of pH. Reactions were terminated by the addition of SDS-loading buffer.

### Parkin cellular assay

Cells were stained as previously reported^52^ with the exception that cells were typically grown in 24-well plastic dishes. Images were captured on the Cellomics ArrayScan VTi platform (Thermo Scientific) using the Target Activation BioApplication to quantify the percentage of cells containing Tom20 mitochondrial staining. Cell fields were imaged using a × 10 objective lens with an average of 250 cells detected per field. Data were collected from at least 2,000 cells per well of a 96-well plate. The readout parameters for the cellular assay were average fluorescence intensity and the percent of cells showing little or no Tom20 staining. The percentage of Tom20 loss relative to full-length wild-type Parkin was calculated by setting full-length wild-type Parkin-induced Tom20 loss after CCCP treatment to 100%. Data presented are representative of three to four independent experiments (error bars represent s.e.m.).

### Statistical analysis

Triple asterisk denotes *P*≤0.005, double asterisk denotes *P*≤0.01 and single asterisks denote *P*≤0.05. The significance levels were determined using the heteroscedastic Student’s *t*-test with two-tailed distribution.

### Detection of cellular Parkin-linked oxyester

Cells were transfected with XtremeGene according to the manufactures protocol (Roche) using untagged full-length Parkin (or mutants) together with HA-ubiquitin with a DNA ratio of 1:10 respectively. Transfections were for 48 h with media exchange and addition of CCCP (10 μM final) after 24 h. Cells were lysed on ice for 30 min in 20 mM HEPES, 150 mM NaCl, 10% glycerol, 1% Triton-X-100, pH 7.2 with EDTA-free complete protease inhibitors (Roche). Lysates were clarified for 10 min at 16,000 *g* in a tabletop microcentrifuge at 4 °C. Protein was quantified using BCA (Thermo Fisher Scientific). To obtain sufficient separation of Parkin and the oxyester-linked Parkin, samples were typically run for 2 h on a 10% Tris-Glycine gel (Invitrogen). For immunoprecipitation of oxyester-linked Parkin, one mg of protein extract (after a one hour preclear with protein A beads alone) was incubated with Protein A resin and HPA1A (5 μg) overnight with rotation at 4 °C. The next day samples were washed 3 × with lysis buffer followed by addition of SDS-loading dye. Reactions were then incubated with NaOH (0.14 mol l^−1^) or buffer control for 20 min at 37 °C before being boiled.

### Mass spectrometry

For LC-MS/MS identification of Parkin-C431-Ub-VS modified peptide tryptic digests of Parkin reacted with HA-Ub-VS were analysed on an ABSciex 5600 qTOF mass spectrometer using a method in which each survey MS scan was followed by MS/MS analysis of the 30 most abundant peaks in the MS spectrum. Identification of peptides was performed using Mascot version 2.4^53^, with 10 p.p.m. for peptide mass tolerance, and 0.1 Da for MS/MS tolerance. To determine the peptide identifications the Uniprot database was searched using oxidation (M+15.9949 Da), deamidation (NQ+0.9840 Da), carbamidomethylation (C+57.0214 Da) and the GG-vinyl sulphone remnant (C+192.0569 Da) as variable modifications.

## Author contributions

The manuscript was written jointly by B.E.R., J.C.L., D.R.A. and T.Y. and J.A.J., B.E.R. and M.V. performed all cellular experiments. B.E.R. performed all biochemical experiments. Structural results were contributed by J.L., E.B., N.Y. and D.R.A; E.B. crystallized the protein and J.C.L. solved the structures. K.C. performed all protein purification. Y.Y. and L.D. carried out protein expression. B.E.R. and D.R.A. devised the Ub-VS experiments. J.B., J.A.J. and B.E.R. devised constructs and Z.Z., Y.Y., S.C., M.V. and B.E.R. made constructs. D.W. and T.S. performed all mass spectrometry experiments. L.N. performed the Arrayscan for Tom20 loss. M.B. and K.R. provided reagent validation. All authors contributed to overall study design and J.A.J. provided the conceptual framework for this project.

## Additional information

**Accession codes:** Coordinates and structure factors have been deposited in the RCSB protein data bank under accession codes 4I1F (Parkin-R0RBR-P223) and 4I1H (Parkin-R0RBR-S223).

**How to cite this article:** Riley, B. E. *et al*. Structure and function of Parkin E3 ubiquitin ligase reveals aspects of RING and HECT ligases. *Nat. Commun.* 4:1982 doi: 10.1038/ncomms2982 (2013).

## Supplementary Material

Supplementary Information Supplementary Figures S1-S8 and Supplementary Tables S1-S2
