# Supplementary material for: Structure and function of Parkin E3 ubiquitin ligase reveals aspects of RING and HECT ligases
Source: Nat Commun. 2013 Jun 17;4:1982. doi: 10.1038/ncomms2982 (PMC3709503; doi:10.1038/ncomms2982)
Supplement: Supplementary Information — Supplementary Figures S1-S8 and Supplementary Tables S1-S2 [file ncomms2982-s1.pdf]

SUPPLEMENTARY INFORMATION

Structure and Function of Parkin E3 Ubiquitin Ligase Reveals Aspects of RING and HECT Ligases

Riley, B.E.[*1], Lougheed, J.C.[*1], Callaway, K.[1], Velasquez, M.[1], Brecht, E.[1], Nguyen, L.[1], Shaler, T.[2], Walker, D.[1], Yang, Y.[1], Regnstrom, K.[1], Diep, L.[1], Zhang, Z.[1], Chiou, S.[1], Bova, M.[1], Artis, R.A.[1], Yao, N.[1], Baker, J.[1], Yednock, T.[1], Johnston, J.A.[+1]

[1] Elan Pharmaceuticals, South San Francisco, CA, 94080, USA
[2] SRI International, Menlo Park, CA, 94025, USA

* These authors contributed equally to this work
[+] To whom correspondence should be addressed, Jennifer Johnston, jenniferjohnston@e3xbio.com

a

b

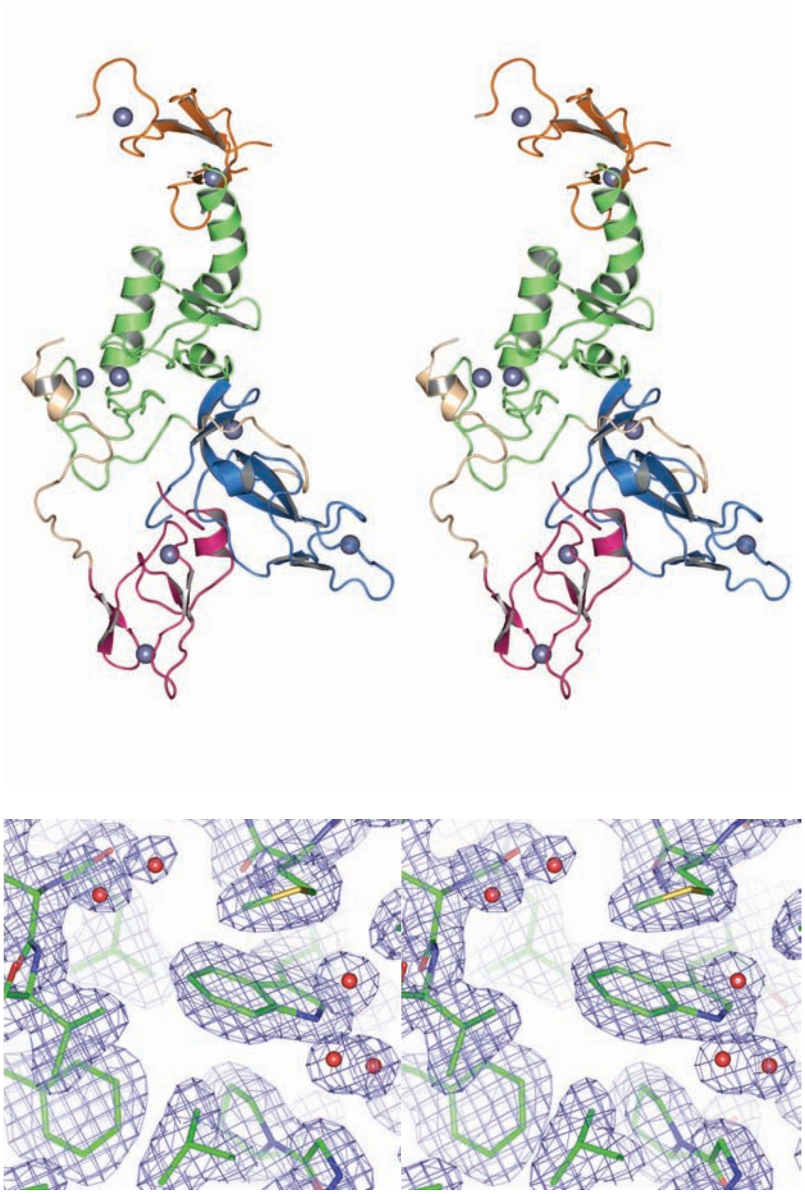

Supplementary Figure S1 Stereo ribbon diagram of Parkin R0RBR-P223 and electron density map of R0:R2.
a, Stero ribbon diagram of Parkin R0RBR **b,** The MAD phased map of Parkin R0RBR P223 is of high quality.
A representative view of the electron density, contoured at 1σ, is shown at the R0:R2 interface. For orientation, Tryptophan 462 is in the middle of the image.

a

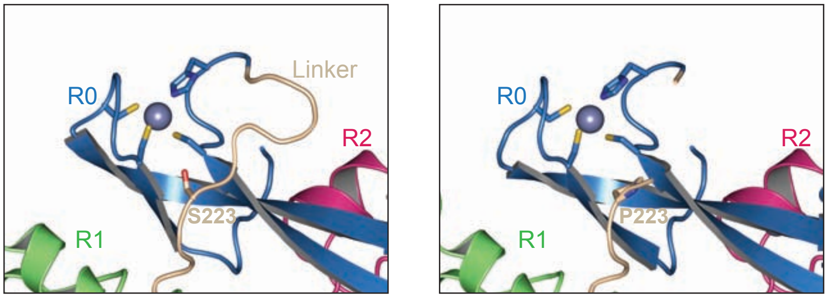

Supplementary Figure S2 Comparison of Parkin R0RBR-S223 and Parkin R0RBR-P223.
a, The loop containing residue 223 is ordered in Parkin R0RBR-S223 while it is disordered in the Parkin R0RBR-P223 structure. The nearby bound Zn site is well ordered in the S223 structure while it appears to have more than one conformation in the P223 structure as indicated by the diffuse electron density and higher crystallographic B-factors for this region. The same dominant conformation was modeled in both structures. The coordination residues for the nearby bound Zn (grey spheres) are shown in sticks.

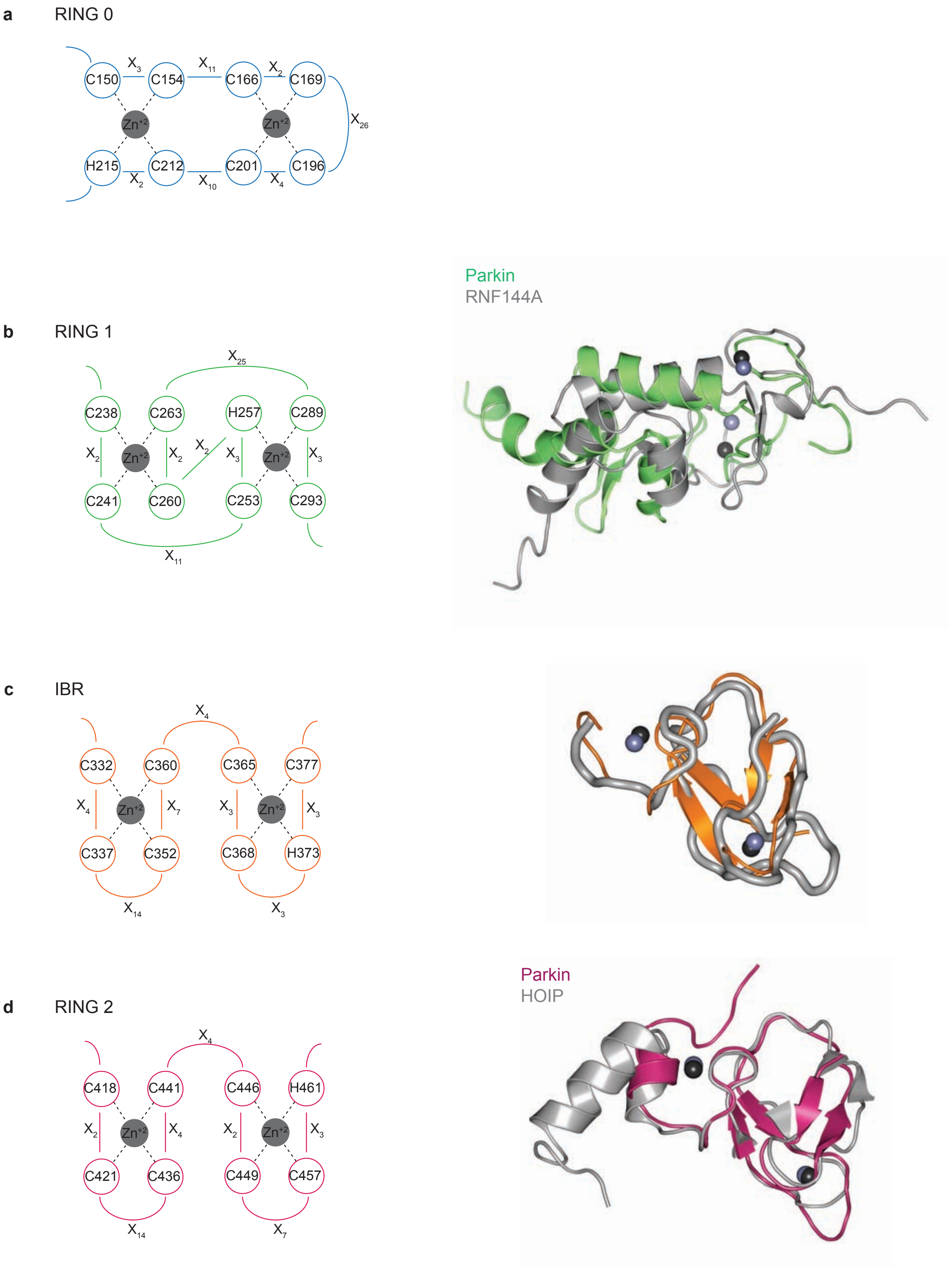

Supplementary Figure S3 Cartoon representations of RING domains and identification of Zn coordinating residues.
a, R0 domain is a previously unobserved fold. **b,** The R1 domains of Parkin (green) and RNF144A (pdb 1WIM, grey) are similar. **c,** The IBR domain from our crystal structure (orange) agrees with the IBR domain solved by NMR (pdb 2JMO, grey). **d,** The Parkin R2 domain (pink) is similar to the NMR structure of the IBR domain of HOIP (pdb 2CT7, grey).

a

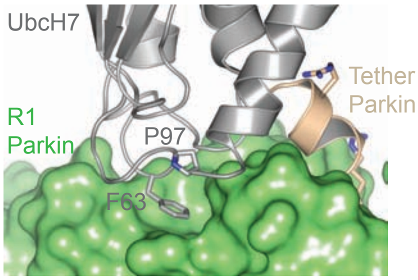

b

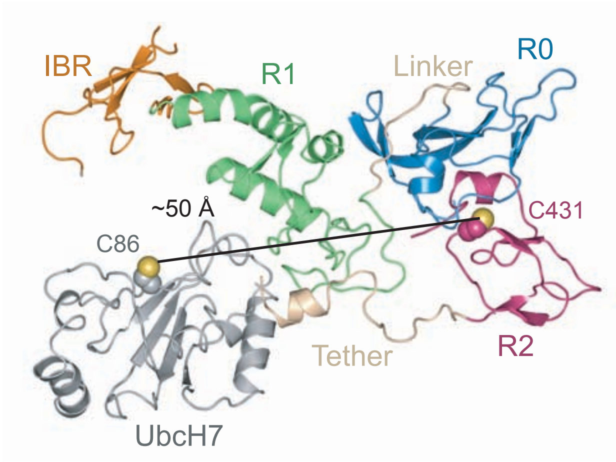

c

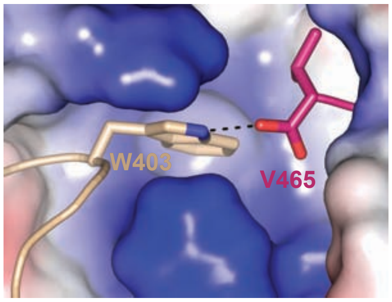

Supplementary Figure S4 Superposition of UbcH7 on Parkin R1 domain.
a, UbcH7 (grey) was positioned on Parkin-R0RBR by superimposing R1 (green) with the RING domain in the structure of c-Cbl bound to UbcH7 (pdb 1FBV) using the pymol align command; c-Cbl is not shown for clarity. There is compatible binding between the hydrophobic grooves of Parkin R1 and UbcH7 (canonical residues involved in E2 binding, F63 and P97 of UbcH7 are labeled), similar to that previously described[47].
b, The catalytic cysteines of UbcH7 and Parkin-R0RBR are > 50 Å apart in our superposition model and the Parkin tether residues 393-395 (beige) overlap with UbcH7 in the superposition, suggesting that they adopt another conformation in E2 bound R1. Conformational changes and/or oligomerization could bring the catalytic cysteines into proximity for transthiolation. **c,** Electrostatic surface representation around W403 demonstrates that the inside of the pocket is hydrophobic.

a

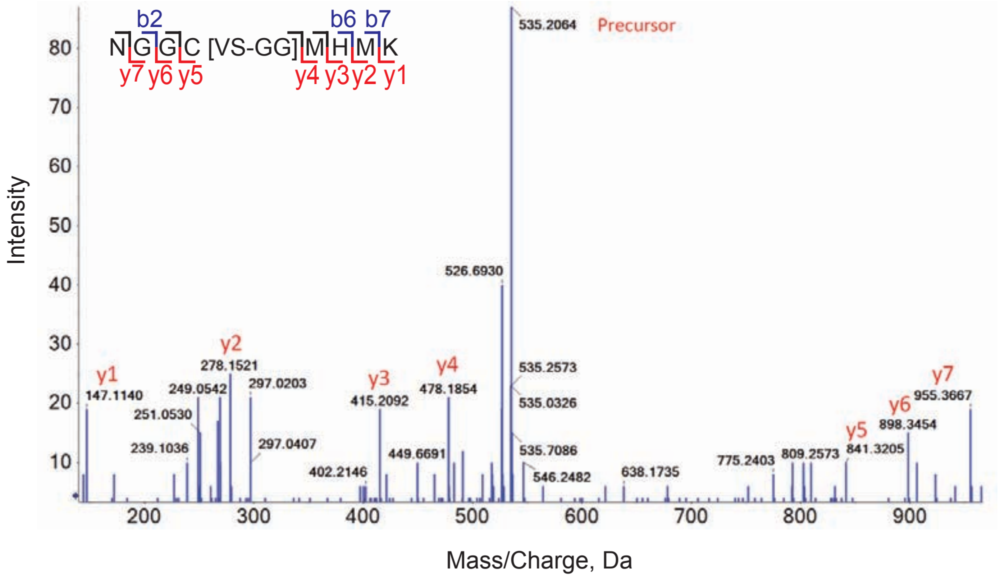

#	b-ions	Seq.	Y-ions	#
1	115.0502	N		8
2	172.0717	G	*955.3617*	7
3	229.0931	G	*898.3402*	6
4	524.1592	C#	*841.3187*	5
5	655.1997	M	*546.2527*	4
6	792.2586	H	*415.2122*	3
7	923.2991	M	*278.1533*	2
8		K	*147.1128*	1

C# = Cys + Vinyl Sulfone-GlyGly

b

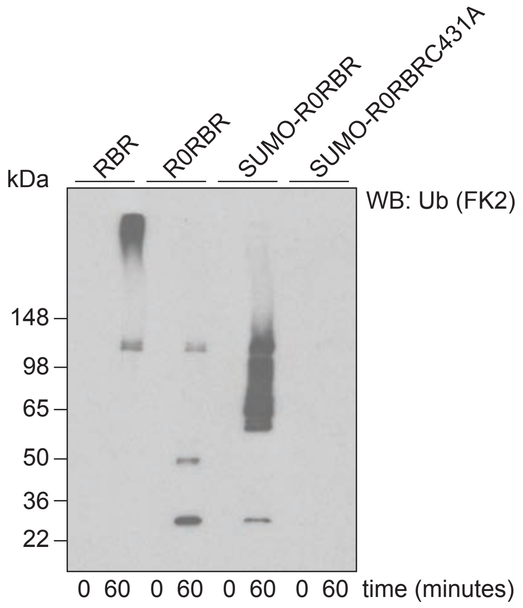

Supplementary Figure S5 Parkin conjugation to HA-UbVS occurs on C431.
a, Mass spectrometry analysis of Parkin HA-UbVS reactions. **b,** Autoubiquitination of various Parkin constructs as monitored with immunoblotting with anti-Ub antibody (FK2).

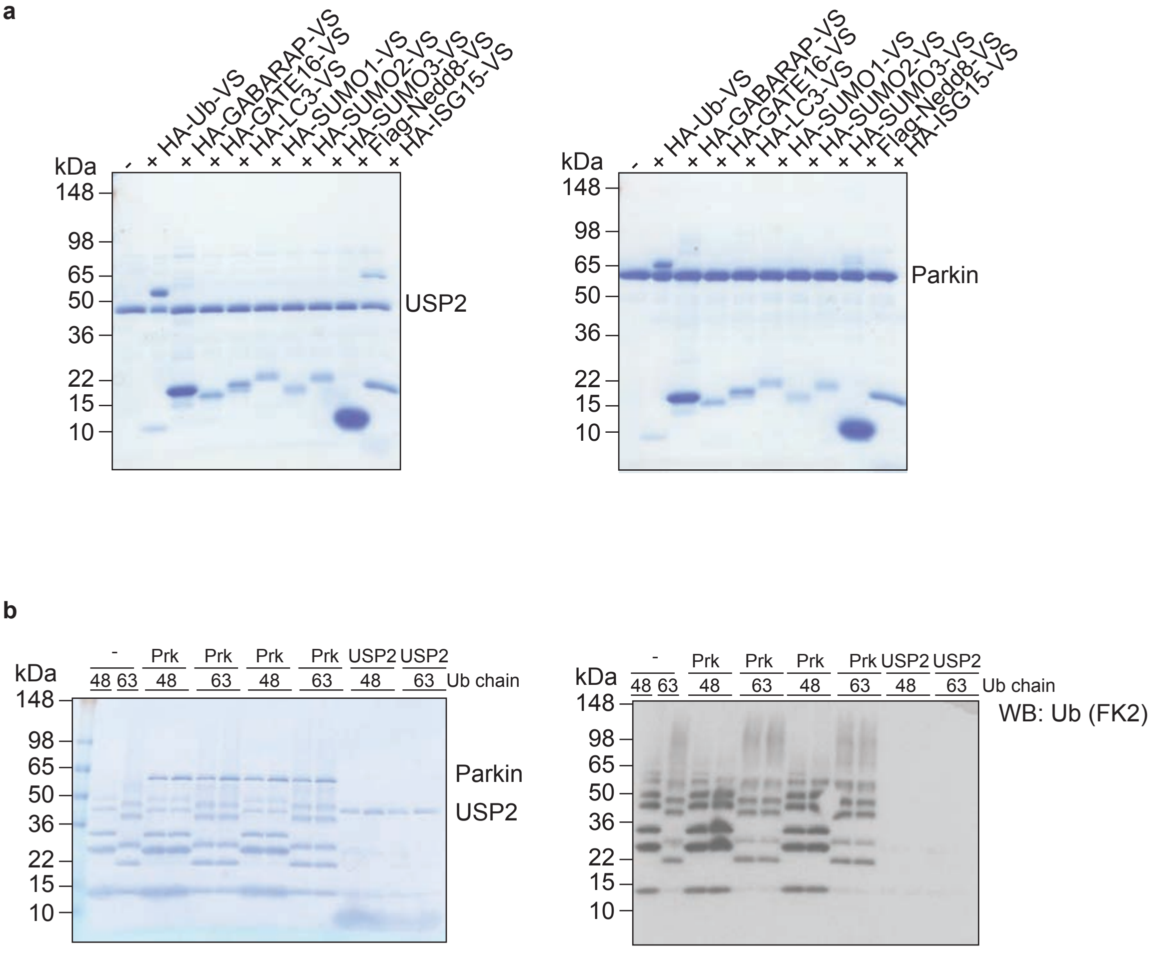

Supplementary Figure S6 Parkin reactivity with HA-UbVS is specific and Parkin is not a deubiquitnating enzyme (DUB).
a, The DUB USP2 reacts with HA-UbVS and HA-Isg15VS (also shown previously[54]), and was used as a positive control. Parkin reacts specifically with HA-UbVS, and to a weak extent NEDD8, and no reactivity with other Ub-like VS probes. **b,** Parkin or USP2 were incubated with pure UbK48 (2-7) or UbK63 (2-7) chains. Reactions were monitored by Coomassie blue or western blot with the Ub antibody FK2. For USP2, Ub chains are completely disassembled whereas Ub chains remained intact when incubated with Parkin.

[54]Catic, A. et al. Screen for ISG15-crossreactive deubiquitinases. *PLoS One* **2**, e679, doi:10.1371/journal.pone.0000679 (2007).

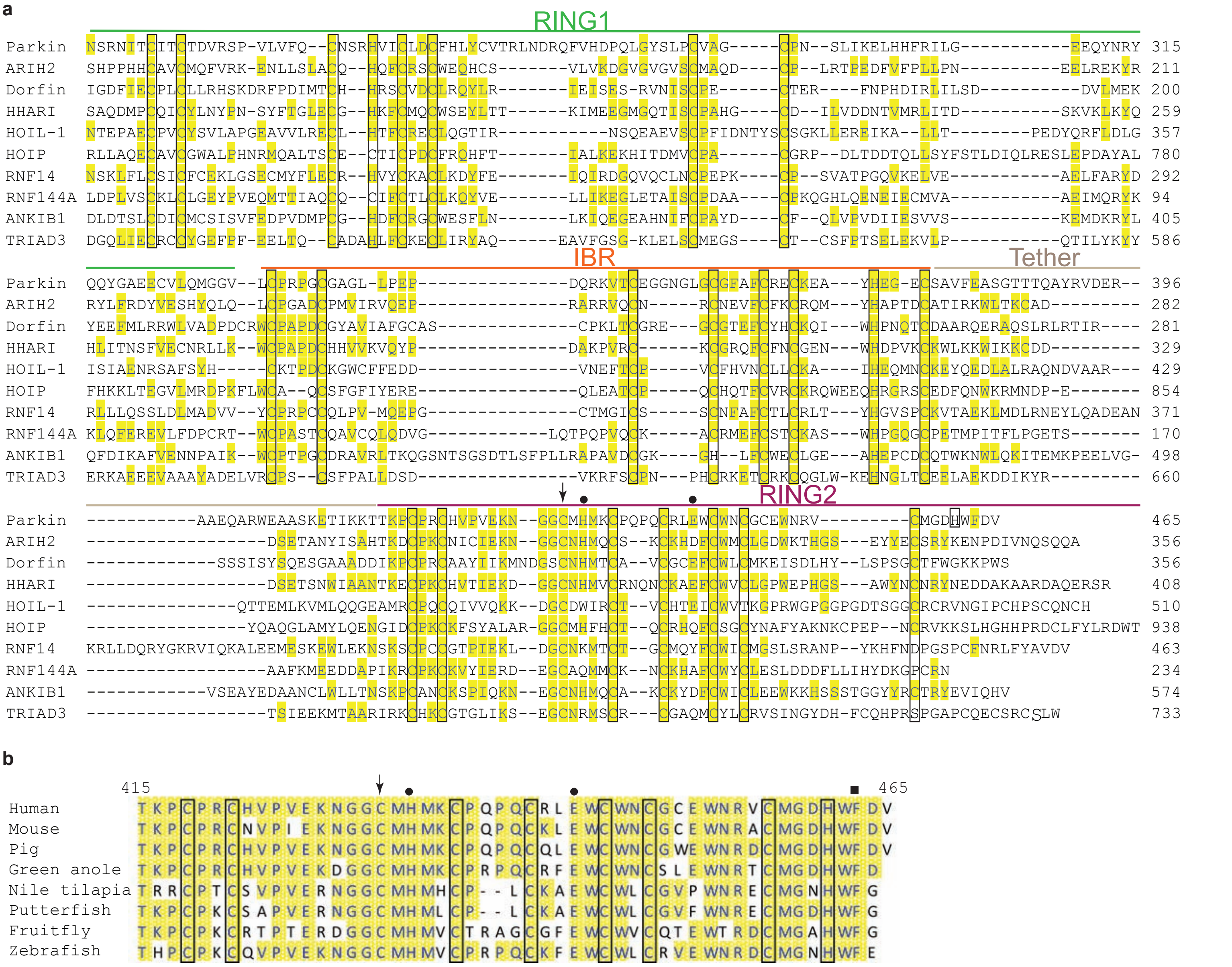

Supplementary Figure S7 Alignment of human RBR proteins and Parkin R2 from different species.
a, The Parkin R1, IBR, and R2 domain boundaries are indicated over the structure and conserved residues are highlighted in yellow and Zn coordination residues are boxed. The catalytic C431 of Parkin is indicated with an arrow, and H433 and E444 are indicated with black circles. HOIL-1 does not have a conserved base at the position corresponding to Parkin H433, and is not catalytic in the LUBAC complex[1]. **b,** Alignment of Parkin R2 from eight different species. The catalytic C431 of Parkin is indicated with an arrow, and H433 and E444 are indicated with black circles. Conserved C-terminal residue F463 involved in the R0:R2 interface is indicated with a black box.

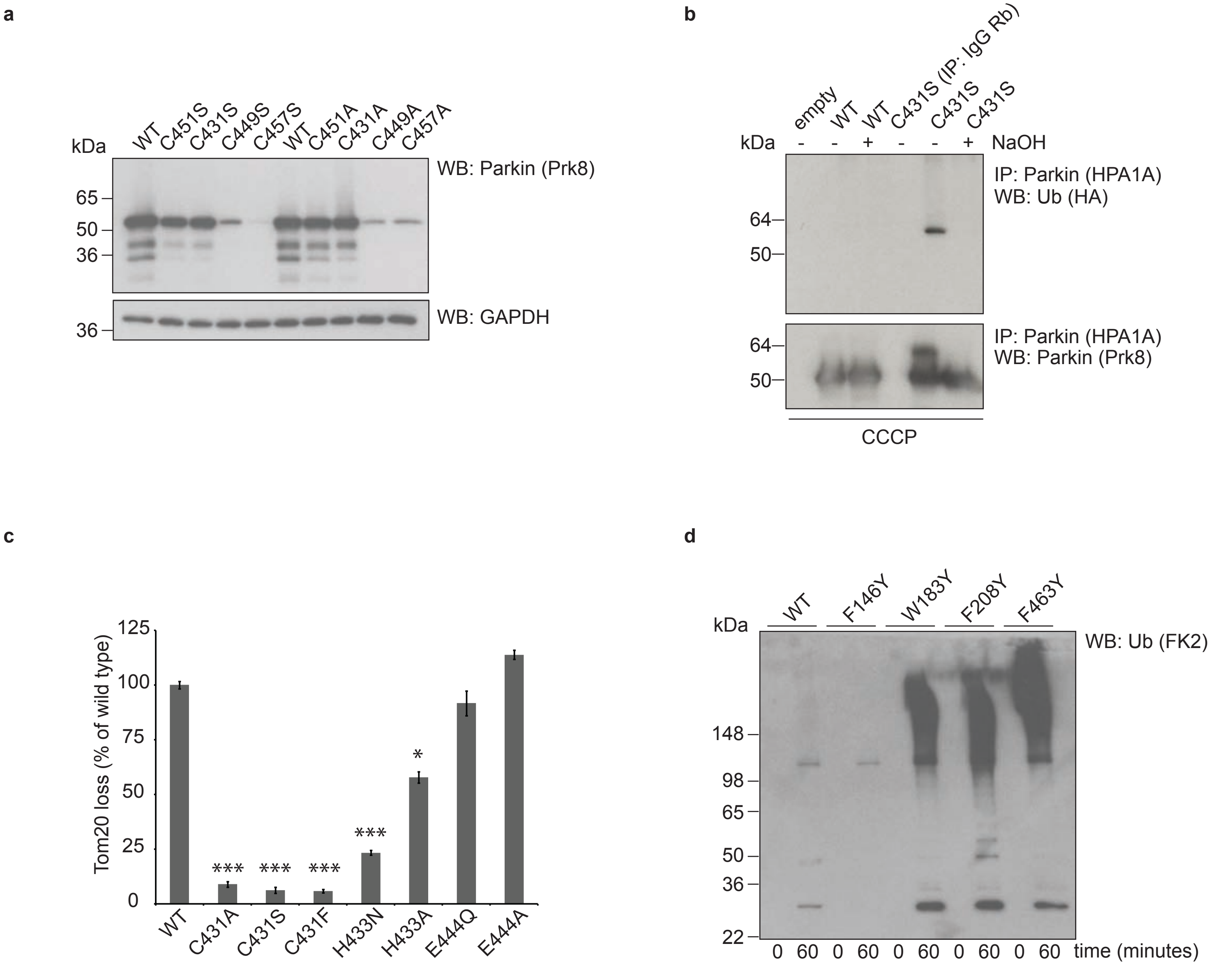

Supplementary Figure S8 Parkin's catalytic machinery.
a, Soluble levels of untagged Parkin cysteine (C) to serine (S) or alanine (A) mutants in transfected HeLa cells. GAPDH was used as the loading control. **b,** Immunoprecipiation (IP) of Parkin followed by western blotting (WB) with HA to demonstrate the ~8 kDa shift is HA-Ub. **c,** Parkin-induced Tom20 loss after CCCP requires H433 but not E444. Data shown is representative of four independent experiments (standard error represents s.e.m.). The significance levels were determined using the heteroscedastic Student's t-Test with two-tailed distribution. Triple asterisk denotes $P < .005$, double asterisk denotes $P < .01$ and single asterisks denote $P < .05$. C431A $P = .002$, C431S $P = .002$, C431F $P = .002$, H433N $P = .003$, H433A $P = .016$. **d,** Parkin R0RBR autoubiquitination activity for various R0:R2 mutants was monitored by immunoblotting with anti-Ub antibody (FK2).

Crystal	Parkin-R0RBR-P223[a]		
**Data Collection**			
Space Group	$C222_1$		
Unit cell: a, b, c (Å)	86.96, 133.2, 65.39		
	HiRes	Peak (Zn)	Remote(Zn)
Wavelength (Å)	1.1159	1.2831	1.2699
Resolution (Å)	44-1.58(1.62-1.58)[b]	50-1.8(1.85-1.8)	50-1.8(1.85-1.8)
Rsym	0.078(0.99)	0.107(-)	0.113(-)
I/δI	11.0(1.6)	14.8(1.0)	15.4(1.3)
Completeness (%)	99.5(99.8)	95.1(63.6)	97.3(70.5)
Redundancy	6.9(6.9)	14.3(10.4)	15.6(11.6)
**Refinement**			
Resolution (Å)	44-1.58		
No. reflections	52033		
Rwork/Rfree[c]	0.205/0.245		
Number of atoms			
Protein	2403		
Ligand/ion	9		
Water	267		
B-factors			
Protein	27.0		
Ligand/ion	26.7		
Water	34.5		
R.m.s. deviations			
Bond lengths (Å)	0.025		
Bond angles	2.26°		

[a] One crystal was used for all data sets. The Peak and Remote data sets were used to determine experimental phases and the final structure was refined against a high resolution (HiRes) data set.

[b] Values in parentheses indicate the highest resolution shell

[c] R_{free} was calculated with a randomly chosen 5% of the data for the P223 structure and this same set was used for the S223 structure

Supplementary Table S1 Data collection and refinement from the crystallography studies of Parkin R0RBR P223.

Crystal	Parkin-R0RBR-S223[a]
**Data Collection**	
Space Group	$C222_1$
Unit cell: a, b, c (Å)	87.11, 133.9, 66.21
Resolution (Å)	17-2.00(2.12-2.00)[b]
Rsym	0.111(0.398)
I/σI	12.1(3.6)
Completeness (%)	82.3(36.3)[c]
Redundancy	6.1(5.5)
**Refinement**	
Resolution (Å)	17-2.00
No. reflections	21783
Rwork/Rfree[d]	0.181/0.215
Number of atoms	
Protein	2405
Ligand/ion	8
Water	263
B-factors	
Protein	20.6
Ligand/ion	17.7
Water	23.3
R.m.s. deviations	
Bond lengths (Å)	0.019
Bond angles	1.84°

[a] One crystal was used

[b] Values in parentheses indicate the highest resolution shell

[c] Completeness was 95.6% to 2.24Å at the edge of the detector

[d]R_{free} was calculated with a randomly chosen 5% of the data for the P223 structure and this same set was used for the S223 structure

Supplementary Table S2 Data collection and refinement from the crystallography studies of Parkin R0RBR S223.
